# The Regulation of Spermatogonial Stem Cells in an Adult Testis by Glial Cell Line-Derived Neurotrophic Factor

**DOI:** 10.3389/fendo.2022.896390

**Published:** 2022-06-03

**Authors:** William W. Wright

**Affiliations:** Department of Biochemistry and Molecular Biology, Johns Hopkins Bloomberg School of Public Health, Baltimore, MD, United States

**Keywords:** GDNF (glial cell line-derived neurotrophic factor), spermatogonial stem cells (SSC’s), sertoli cell-only syndrome, mature testis, male infertility, sertoli cell

## Abstract

This review focuses on the *in vivo* regulation of spermatogonial stem cells (SSCs) in adult testes by glial cell line-derived neurotrophic factor (GDNF). To study adult mouse testes, we reversibly inhibited GDNF stimulation of SSCs *via* a chemical-genetic approach. This inhibition diminishes replication and increases differentiation of SSCs, and inhibition for 9 days reduces transplantable SSC numbers by 90%. With more sustained inhibition, all SSCs are lost, and testes eventually resemble human testes with Sertoli cell-only (SCO) syndrome. This resemblance prompted us to ask if GDNF expression is abnormally low in these infertile human testes. It is. Expression of FGF2 and FGF8 is also reduced, but some SCO testes contain SSCs. To evaluate the possible rebuilding of an SSC pool depleted due to inadequate GDNF signaling, we inhibited and then restored signaling to mouse SSCs. Partial rebuilding occurred, suggesting GDNF as therapy for men with SCO syndrome.

## Introduction

Spermatogonial stem cells (SSCs) are the foundation of male fertility. Preserving this foundation requires that their replication sustains a stem cell pool of normal size and also produces sufficient numbers of differentiating progenitor spermatogonia to ensure continuous production of the large numbers of sperm required for fertility (1). As with all other stem cells, SSCs reside in a special physiological environment or niche that in the testis is created by testicular somatic cells, including Sertoli and peritubular myoid cells (2, 3). These somatic cells secrete numerous growth factors and cytokines that regulate SSC replication and differentiation. Glial cell line derived neurotrophic factor (GDNF) was the first growth factor demonstrated to be essential for the normal function of the SSC niche (4). Prepubertal GDNF^+/-^ mice do not generate the numbers of SSCs necessary to sustain spermatogenesis in the adult. However, until recently, the role of GDNF in a normal adult testis had not been evaluated.

Given this gap in our knowledge and the obvious importance of SSCs to human male fertility, we focused our research on the role of GDNF in the regulation of SSCs within in a mature mouse testis. Results of those studies then prompted us to ask whether a deficit in GDNF expression might contribute to the most severe form of human nonobstructive azoospermia, Sertoli cell-only (SCO) syndrome. This syndrome is characterized by the apparent absence of spermatogenic cells in histological sections of testes (5). This review summarizes the results of our studies that collectively address three hypotheses:

Hypothesis 1: GDNF is essential for sustaining SSCs in an adult mouse testis. We predict that inhibition of GDNF signaling causes numbers of these stem cells to rapidly decline, due to their differentiation and cessation of self-renewing replication.

Hypothesis 2: Sertoli cells in human SCO testes express abnormally low levels of GDNF.

Hypothesis 3: A mouse SSCs pool that has been partially depleted due to inhibition of GDNF signaling will rebuild if GDNF signaling is restored.

This review also places our results in the context of current literature on the presence of multiple subtypes of SSCs in a mature testis, the transcriptomes of human SSCs and Sertoli cells and analyses by other laboratories of human SCO testes. Building on this summary, we end with a proposal for a potential new therapy for some men with SCO syndrome. As this proposal for human therapy is founded on our analysis of the restoration of a depleted pool of mouse SSCs, it is appropriate to begin with a brief summary of similarities and differences between these stem cells in mice and men (6). Obviously, the first important similarity is that mice and men contain SSCs, as defined by their abilities to survive and replicate when transplanted into a germ cell-deficient mouse testes (7, 8). We acknowledge that transplanted mouse but not human SSCs generate the entire spermatogenic lineage when transplanted into mouse testes (7). Hermann and colleagues attributed the results with human SSCs as being due to the evolutionary distance between mice and men (7). This suggestion is reasonable since testes of another primate species, Rhesus macaques, contains SSCs as defined by their ability to seed the entire spermatogenic lineage when transplanted into germ cell-deficient monkey testes (9). The second similarity is that mouse and human SSCs express many of the same stem cell markers (7). These include: GFRA1, the ligand binding subunit of the GDNF receptor (10), UTF1, a stimulator of self-renewing stem cell replication (11), LIN28 a regulator of stem cell pluripotency and metabolism (12), ZBTB16, a transcription factor necessary for preservation of SSC stemness (13), and ID4, a dominant-negative inhibitor of basic helix-loop-helix transcription factors (14). Importantly, SSCs of neither species express KIT, the receptor for Kit ligand, a stimulator of spermatogonial differentiation (15).

There is, however, a major difference between mouse and human SSCs. As discussed in detail in two excellent reviews by Orwig and co-workers, numbers of SSCs per gram testis are much higher in humans than in mice (6, 7). It has been proposed that the higher number of human SSCs compensates for fact that human spermatogonia replicate fewer times before the start of meiosis (7). Based on the data presented in those 2 reviews we estimate that numbers of SSCs per gram testis are 4-fold higher in men than mice. This estimate is consistent with our analyses; numbers of GFRA1^+^ spermatogonia per mm^2^ tubule surface are 4.2-fold higher in men than mice (5, 16).

## Hypothesis 1: GDNF Is Essential for Sustaining SSCs in an Adult Mouse Testis

### The Experimental Model

**Figure 1:**
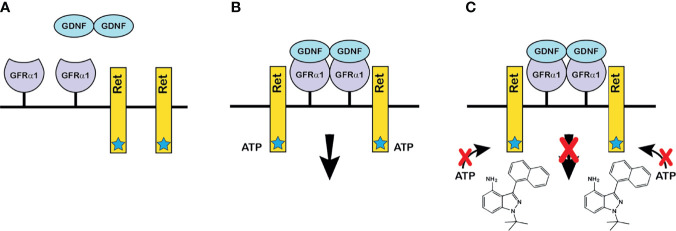
The chemical-genetic approach to reversible inhibition of GDNF signaling *in vivo*. **(A)** GDNF and the components of the GDNF receptor: GDNF is secreted as a disulfide-bonded dimeric protein (17). GFRα1 is the ligand binding subunit of the GDNF receptor, which is linked to the plasma membrane by glycosylphosphatidylinositol (18). Ret is the receptor’s protein kinase subunit. The star on Ret denotes the enlarged ATP binding pocket of Ret(V805A). **(B)** The GDNF receptor-ligand complex and the initiation of intracellular signaling: Dimeric GDNF cross-links two GFRα1 receptor subunits, which then recruit two Ret receptor subunits. Formation of the ligand-tetrameric receptor complex stimulates Ret kinase activity, and Ret-bound ATP donates a phosphate to tyrosine residues on the intracellular domain of Ret. Phosphorylation initiates an intracellular signaling cascade. The V805A mutation has no effect on normal Ret kinase activity. **(C)** How the chemical-genetic approach works: The bulky ATP-competitive inhibitor, 1NAPP1 (structure shown on figure) binds with high affinity to the ATP binding pocket of Ret(V805A). Consequently, 1NAPP1 prevents RET phosphorylation. Daily injections of 1NAPP1 are sufficient to inhibit GDNF signaling but signaling is restored when injections cease.

A prerequisite for studying the role of GDNF in a normal mature mouse testis was that experiments start with animals whose testes contained a full complement of SSCs and differentiated spermatogenic cells. Meeting this prerequisite required that GDNF signaling to SSCs be altered only in the adult animal. Furthermore, to test our third hypothesis, this alteration must be reversible. Consequently, we developed a novel chemical-genetic approach that allowed specific and reversible inhibition of stimulation of SSCs by GDNF (**Figures 1A–C**). Our approach had two components: First, we developed a line of mice with a single amino acid mutation (V805A) in Ret, the kinase subunit of the GDNF receptor (19). This mutation enlarged the size of the ATP binding pocket of Ret, without affecting normal RET kinase activity. However, this enlargement enabled Ret(V805A) to bind a bulky, high affinity ATP competitive inhibitor, 1NAPP1-HCl (hereafter called 1NAPP1). Ret(V805A) mice were normal and fertile. However, injection of these mice with 1NAPP1, blocked the ability of GDNF to stimulate its target cells (**Figure 1C**). Surprisingly, while RET is expressed in many adult mouse organs (https://www.gtexportal.org/), we only detected an effect of 1NAPP1 in the Ret (V805A) mouse testis (19). Of equal importance, normal Ret signaling was restored to any remaining SSCs when injection of the inhibitor ceased.

### First Test of Hypothesis 1

Our first test of the hypothesis that GDNF is essential for sustaining SSC s in an adult testis took advantage of the fact that the sustained loss of SSCs from a seminiferous tubule is followed by the sequential loss of all remaining spermatogonia, of spermatocytes and then of spermatids. Eventually maturation depletion results in a tubule devoid of all germ cells. It follows that the higher the percentage of germ cell-deficient tubules, the lower the numbers of SSCs at the time testes are collected for analysis. In several experiments, we injected between three and five Ret(V805A) mice with 1NAPP1 once a day for 7 to 30 days and then waited 35 or 60 days for maturation depletion to occur. We then prepared 1-micron cross sections from 4 to 6 different areas of each testis, and determined the percentage of tubule cross sections without germ cells, including spermatogonia. Results demonstrated that this percentage increased as the duration of inhibited GDNF signaling increased (**Table 1**). Importantly, when mice were treated for 11 and 30 days, 97% and 100% of tubules, respectively lacked all spermatogenic cells (**Table 1**). (We examined a total of 1200 (11 days) and 1500 (30 days) tubule cross sections in that experiment.) In contrast, when mice were treated for 7 or 9 days, about 5% and 47% of tubules, respectively, lacked germ cells. As SSCs are the foundational spermatogenic cells, and as after 30 days of treatment, all seminiferous tubules we examined were devoid of spermatogenic cells, we conclude that GDNF is essential for maintenance of SSCs in an adult mouse testis. However, because of the length of time between treatment and analysis, those experiments did not reveal whether inhibition of GDNF signaling caused rapid stem cell loss.

**Table 1:** Effect of the duration of treatment of mice with 1NAPP1-HCl on the percent of Seminiferous Tubules that lack all Spermatogenic Cells 35 or 60 days after treatment.

Treatment	1NAPP1-HCl	Days From	Number of	Total No.	% Tubules	Citation
Duration	mg/kg body	Treatment	Treated	Tubules	Lacking	
(days)	weight	to Tissue	Animals in	Examined in	All Germ	
		Collection	Experiment	Experiment**	Cells*	
30	62.5	35	5	1500	100	6
11	62.5	35	4	1200	97	6
9	43.7	36	3	900	50	13
9	43.7	60	3	900	45	22
7	43.7	35	3	900	5	13

*1-micron histological sections were prepared from 4-6 different areas of each testis. 300 tubules from each testis were examined.

**Numbers of animals X 300 tubules analyzed/animal.

### Second Test of Hypothesis 1

**Figure 2:**
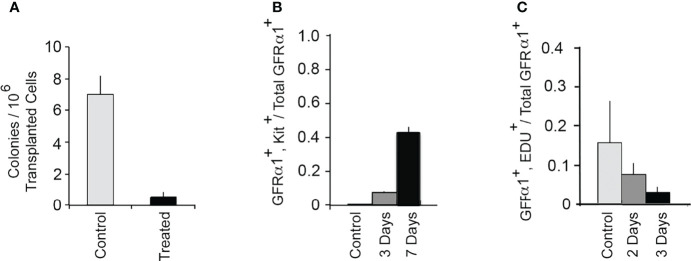
GDNF is essential for sustaining SSCs in an adult mouse testis. **(A)** Effect of inhibition of GDNF signaling for 9 days on numbers of transplantable SSCs. The Ret (V805A) mice used in this study were heterozygous for bacterial β−galactosidase (*Rosa 26*) and for *Id4-GFP*. Adult mice were injected daily for 9 days with 1NAPP1 (treated) or with vehicle (control). Two to four days after the last injections, germ cells were isolated from these mice, transplanted into testes of germ cell-deficient mice, and testes analyzed 2 months after transplantation. Numbers of transplanted SSCs were estimated by enumerating colonies of Rosa 26^+^ spermatogenic cells in each testis. Data (mean + SEM) demonstrate that inhibition of GDNF signaling for 9 days results in loss of 90% of transplantable SSCs. Data are from: (16). **(B)** Inhibition of GDNF signaling results in rapid differentiation of SSCs. In this experiment we defined SSCs as GFRα1^+^, A single (A_s_) spermatogonia, and we used their co-expression of Kit to identify differentiating cells (1). Adult Ret(V805A) mice were treated with vehicle or with 1NAPP1 for 3 or 7 days, seminiferous tubules collected 24 hours after the last injection, and tubules processed for identification of both GFRα1^+^ and GFRα1^+^, Kit^+^ A_s_ spermatogonia. Data (mean + SEM) are presented as numbers of GFRα1^+^, Kit^+^ A_s_ spermatogonia divided by total numbers of total GFRα1^+^A_s_ spermatogonia. Results demonstrate that in control mice, only 0.0008 of all GFRα1^+^, A single (A_s_) spermatogonia express Kit. However, after 3 and 7 days of treatment with 1NAPP1, 0.08 and 0.42, respectively of all GFRα1^+^ A_s_ spermatogonia, expressed Kit. Data are from: (20). **(C)** Acute inhibition of GDNF signaling causes a rapid decrease in replication of SSCs in normal, mature testes. Ret(V805A) mice were injected with 1NAPP1 for 2 or 3 days, and also with the thymidine analogue, EdU on the last day of treatment. Tubules were collected 24 hrs. later and processed for detection of GFRα1 and EdU incorporation. Data are expressed as fraction of all GFRα1^+^, A_s_ spermatogonia, that had incorporated EdU^+^. Results (mean + SEM) demonstrate that inhibition of GDNF signaling results in a rapid decrease in SSC replication. After 3 days, this replication was reduced to 19% of control. Data are from: (20).

To address whether inhibition of GDNF signaling caused rapid loss of SSCs, we injected mice for 9 days and used a functional test for SSCs, their ability to seed spermatogenesis when transplanted into a germ cell-deficient testis. Transplantation of 1NAPP1- and vehicle injected animals occurred 2-4 days after the last injection. Two months later, we enumerated colonies of spermatogenic cells in the testes that received the transplants. As shown in **Figure 2A**, inhibition of GDNF signaling for 9 days reduced numbers of transplantable SSCs to 10% of control. Thus, during a 9-day period, almost all SSCs depend on GDNF to maintain their stemness. These results plus those obtained in the first tests of hypothesis 1 support the conclusion all SSCs are GDNF-dependent at some time during a 30-day period.

We acknowledge that other studies have identified SSCs that do not express GFRA1, the ligand binding subunit of the GDNF receptor. Cells that do not express the GDNF receptor are by definition GDNF-independent. Some of the evidence for GDNF-independent SSCs is as follows: First, some transplantable SSCs in a mature mouse testis do not express GFRA1 (21). Second, 5-10-day old mouse testes contain a subset of SSCs that can be propagated *in vitro* in a GDNF-independent, FGF2-dependent manner. These cells seed spermatogenesis when transplanted into germ cell-deficient testes. Third, there is considerable heterogeneity in expression of markers of SSCs within a population of highly undifferentiated mouse spermatogonia, a subset of which are SSCs (22). Some of these cells do not express GFRA1 but do express other stem cell markers (6).

In comparing results demonstrating that some SSCs do not express the GDNF receptor with our conclusion that all SSCs are at some time GDNF-dependent, it is important to keep in mind that the demonstration of GFRA1^-^ SSCs represents a “snapshot” of SSCs at one point in time. We examined the consequences of inhibiting GDNF signaling over an extended period. We demonstrated a duration-dependent effect of 1NAPP1-treatment, not only on numbers of GFRA1^+^ A single (A_s_) spermatogonia but also on numbers of A_s_ cells that expresses a different SSC marker, ZBTB16 (19). These A_s_ spermatogonia are thought to encompass both SSCs and the most undifferentiated of progenitor spermatogonia (1). When mice were injected for 11 consecutive days with 1NAPP1, numbers of ZBTB16^+^ cells decreased to 12% of control. Thus, most ZBTB^+^ spermatogonia are GDNF-dependent some time during that 11-day period. Furthermore, it was reported that when GFRA1^-^ cells are transplanted, some transplanted cells began to express GFRA1 (21). It was proposed that niche factors stimulate GFRA1^-^ SSCs to express GFRA1. This is consistent with the proposals of both Guo et al. (23), and of Sharma et al. (24) that SSCs exist in metastable states that allow their adaption to a dynamic stem cell niche. Thus, we propose than an individual SSC may be GDNF-dependent at one point in time, but not at another. Finally, we acknowledge that the isolation and passaging of FGF2-dependent, GDNF-independent transplantable SSCs is experimentally more than one snapshot in time. However immature testes were the source of these SSCs, and the transcriptomes of these cells differ from the transcriptomes of SSCs in adult mouse testes (25). Thus, our experiments differ in a significant way from those that conclude that some SSCs are GDNF-independent. We have examined the effects of inhibition of GDNF signaling over time, not just at one time. Furthermore, we have studied SSCs in mature testes, not stem cells isolated from immature testes.

### Inhibition of GDNF Signaling Causes Differentiation of SSCs and Suppresses Their Replication

There is abundant evidence that *in vitro* GDNF suppresses differentiation and stimulates replication of SSCs, but evidence that this was true *in vivo* was lacking when we began our experiments (3, 8). Thus, we tested whether GDNF suppresses differentiation and stimulates replication of SSCs within a mature mouse testis (20). We defined SSCs morphologically, as GFRA1^+^ A single (A_s_) spermatogonia We used the expression of Kit as a marker of stem cell differentiation, and incorporation of the thymidine analogue, 5-ethynyl-2’-deoxyuridine (EdU), to identify replicating cells. To examine SSC differentiation, we treated Ret(V805A) mice for 3 or 7 days with 1NAPP1 or vehicle and stained cells in whole mounts of seminiferous tubules for both GFRA1 and Kit. Results demonstrated that in controls, fewer then 1% of the GFRA1^+^ A_s_ spermatogonia expressed Kit. However, after 3 and 7 days of treatment, 8% and 40% of these cells, respectively, expressed this differentiation marker (**Figure 2B**).

To test if GDNF is an acute regulator of SSC replication, mice were injected with 1NAPP1 for 2 or 3 days and with EdU on the last day of treatment. Tubules were collected and analyzed 24 hours later. Results showed that inhibition of GDNF signaling for 2 or 3 days decreased SSC replication to 44% and 19% of control, respectively. (**Figure 2C**). However, consistent with these cells’ long cell cycle times, inhibition of GDNF signaling for 2 or 3 days did not decrease cell numbers (20).

Taken together, our results support the hypothesis that GDNF is essential for sustaining SSCs in a normal adult mouse testis. Moreover, this growth factor suppresses SSC differentiation and acts as an acute regulator of the replication of SSCs in a normal adult testis.

## Hypothesis 2: Sertoli Cells in Human SCO Testes Express Abnormally Low Levels of GDNF

**Figure 3:**
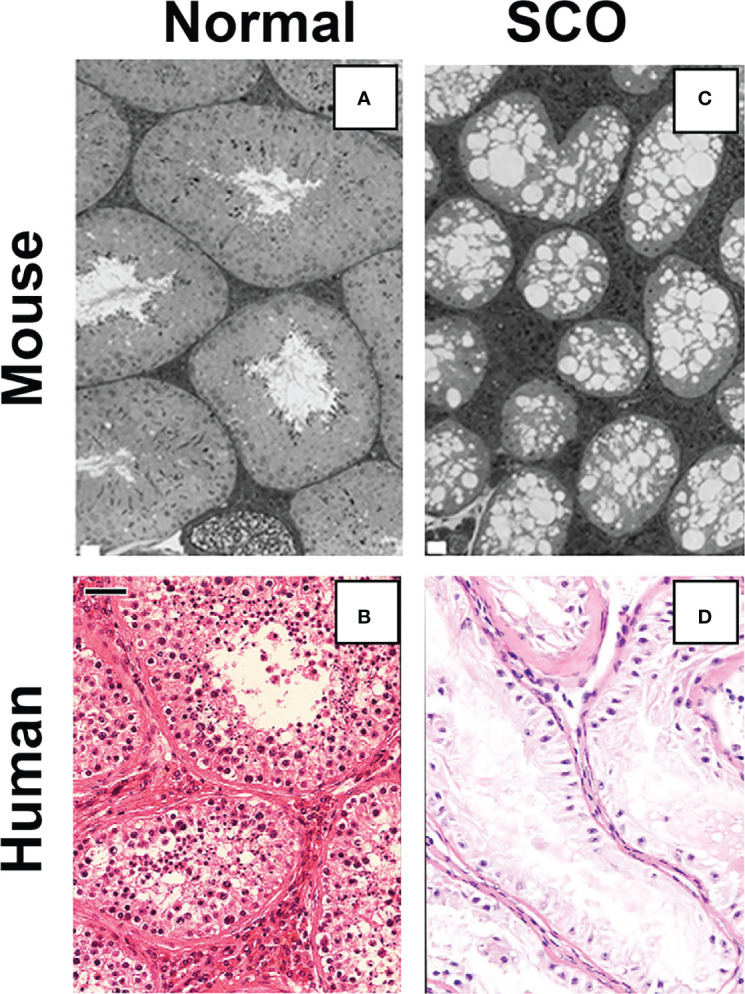
A comparison of the testicular histology of control **(A)** and treated Ret(V805A) mice **(C)** with the histology of normal **(B)** and SCO **(D)** human testes. No spermatogenic cells are evident in the image of testes from treated mouse testis or the image of a human SCO testis. Ret(V805A) mice were injected for 30 days with vehicle **(A)** or with 1NAPP1 **(C)** for 30 days and testes collected 35 days later. Mouse testes were fixed with glutaraldehyde, embedded in epon and 1-micron thick sections stained with Toluidine blue. Sections of human testes were prepared from biopsies collected as part of standard clinical care. Five-micron thick sections were stained with hematoxylin and eosin and all patient identifiers were removed before sections became available for microscopic analysis. The white bars on panels **(A)** and **(C)** are equal to 20 microns on the original section. The black bar on panel **(B)** is equal to 40 microns. Micrographs are from: (5, 19).

The results from our tests of Hypothesis 1 demonstrated that inhibition of GDNF signaling for 11 days caused loss of SSCs and, subsequently, all spermatogenic cells from almost all seminiferous tubules of mature Ret(V805A) mice. Consequently, the testicular histology that the mice developed closely resembled that of human SCO syndrome (**Figure 3**). However, it should be noted that testes of 15%-20% of men with SCO syndrome contain one or more seminiferous tubules with focal areas of active spermatogenesis, allowing sperm retrieval by microdissection testicular sperm extraction (micro-TESE) (26, 27). As loss of mouse germ cells in the Ret(V805) mice resulted from inhibition of GDNF signaling, and as Sertoli cells are the sole and a major source of GDNF in rats and mice, respectively (2, 28), we predicted that Sertoli cells in human SCO testes expressed abnormally low levels of GDNF (5). This prediction was consistent with a preliminary report that cultured Sertoli cells isolated from 2 human SCO testes contained less GDNF mRNA than Sertoli cells obtained from testes of patients with active spermatogenesis. The SCO Sertoli cells also contained significantly lower levels KITL mRNA, a growth factor which stimulates progenitor spermatogonia to differentiate into Type A spermatogonia (29, 30).

### First Test of Hypothesis 2

**Figure 4:**
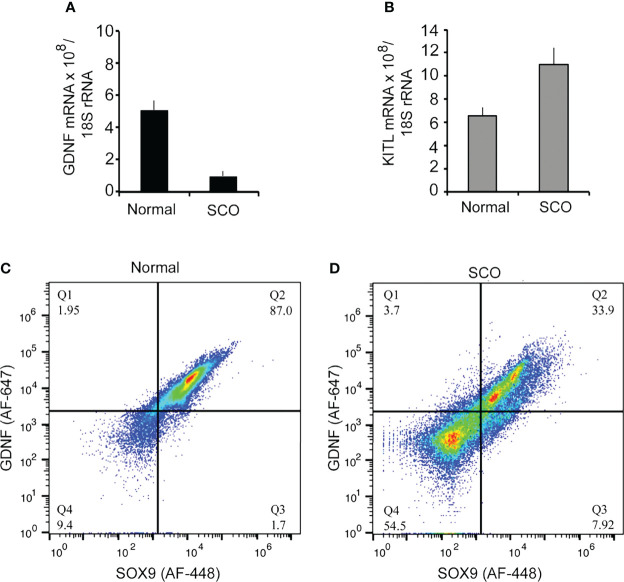
A comparison of the expression of GDNF, and KITL by normal and SCO human testes. RNA was extracted from testis biopsies and expression of GDNF mRNA **(A)**, and KITL mRNA **(B)** assayed by real-time PCR. Data were normalized for βactin mRNA in each sample. GDNF mRNA expression was 5.2-fold higher in normal testes than in SCO testes, while KITL mRNA expression was 1.7-fold higher in SCO testes than in normal testes. **(C, D)** Fluorescence-activated cell sorting was used to determine if human Sertoli cells express GDNF and if this expression is significantly reduced in SCO testes. Single cell suspensions were prepared from biopsies of human normal **(C)** and SCO testes **(D)** and cells incubated with fluorochrome-labeled antibodies for GDNF and the human Sertoli cell specific marker, SOX-9 (23). Data are presented as relative amounts of GDNF and of SOX 9 in each cell. Results, which are representative of 5 independent experiments, demonstrate that normal testes contain a single GDNF-expressing population of Sertoli cells. However, SCO testes contain two populations of Sertoli cells, and the predominant population contains less GDNF than cells in normal testes. Results are from: (5).

We first tested hypothesis 2 by comparing GDNF mRNA levels in normal and SCO human testes (5). Results showed that GDNF mRNA levels were 5.2-fold lower in SCO testes (**Figure 4A**). However, in contrast to the previous report (29), we detected significantly elevated amounts of KITL mRNA in SCO testes (**Figure 4B**). As Sertoli cells are the only source of KITL in a human testis (31), we propose that while we quantified this transcript in testis samples, our results reflect KITL mRNA expression by Sertoli cells. Therefore, we suggest that the previous report of diminished KITL mRNA in SCO Sertoli cells may be due to changes in gene expression caused by the culture conditions.

### Second Test of Hypothesis 2

We next used FACS to determine if Sertoli cells in SCO testes contain markedly reduced levels of GDNF (5). Single cell suspensions were prepared from normal and SCO testes, and cells were immunolabeled for GDNF and for SOX9, a specific marker of human Sertoli cells (23, 32) (**Figure 4C**). Results of 5 independent experiments demonstrated that in a normal human testis, GDNF is produced by a single population of Sertoli cells. Sertoli cells were also the only source of GDNF in SCO testes. However, in SCO testes we identified two different Sertoli populations based on GDNF content (**Figure 4D**). The content of the smaller population was like Sertoli cells in normal testes, but the GDNF content of the predominant population was substantially lower. Thus, in SCO testes most but not all Sertoli cells express abnormally low GDNF levels. This deficit in Sertoli cell function results in GDNF concentration of SCO testes to be only 30% of normal (5).

A recent report from Zhao et al. (32) supports our conclusion that SCO testes contain two populations of Sertoli cells. Results from single cell sequencing led the authors to conclude that SCO testes contain one population of Sertoli cells that is similar to healthy immature Sertoli cells, while the other population is similar to Sertoli cells that have begun to mature. The authors also demonstrated that culturing SCO Sertoli cells in the presence of a Wnt signaling inhibitor resulted in transcriptomes of those cells becoming more like those of mature Sertoli cells. Therefore, they concluded that the dysfunction of Sertoli cells in SCO testes is an intrinsic characteristic to those cells and is not due lack of stimulus from spermatogenic cells, with which Sertoli cells normally interact.

While we are fascinated by the data presented by Zhao et al. (32), we consider their conclusion premature, for there is abundant evidence in rodents and in humans of extensive morphological interactions between Sertoli and spermatogenic cells (33). Furthermore, proteins, genes and pathways, that are molecular bases for these interactions have been identified by studying rats and mice. For example, formation of the blood-testis barrier is considered an important milestone in Sertoli cell maturation (34). However, when a mature seminiferous epithelium experiences sequential loss and restoration of spermatogenic cells, its blood-testis barrier is disassembled and later reformed (16). Furthermore, spermatogenic cells have a profound effect on gene expression by mature rodent Sertoli cells. We identified 198 genes whose expression by rat Sertoli cells waxed and waned from 4 to 900-fold as adjacent germ cells progressed through the stages of the cycle of the seminiferous epithelium (35). The rat cathepsin L (CTSL) gene has proven an excellent model with which to understand how germ cells regulate Sertoli cell gene expression. Stage specific CTSL expression is controlled by sequential stimulatory and inhibitory signals from germ cells, which regulate transcription *via* transcriptional activators and repressors within the CTSL gene promoter (36–39). We acknowledge that as of this date, no one has investigated potential interactions between human germ cells and Sertoli cells. However, Sertoli cells and spermatogenic cells of all mammalian species are organized similarly within the seminiferous epithelium, and developing spermatogenic cells in all mammals translocate along the surface of Sertoli cells in a similar manner. Furthermore, as occurs in all other mammals, the human spermatogonia, spermatocytes and spermatids adjacent to the same Sertoli cell mature synchronously and progress through of the stages of the cycle (33, 40). It therefore seems probable that in a fertile human testis, germ cells significantly affect Sertoli cell gene expression. It follows that an absence of germ cells may be one reason that the transcriptomes of Sertoli cells in SCO testes differ from Sertoli cells in normal testes.

### Some SCO Testes Contain SSCs

When we measured GDNF and KITL mRNA levels in normal and SCO human testes, we also measured DDX4 mRNA, a specific human germ cell marker (41). Surprisingly, DDX4 mRNA was detectable in all SCO testes, albeit at very low levels, which suggested that some SCO testes contain SSCs (5). To explore this possibility, we used RNAseq to define the transcriptomes of 4 normal and 7 SCO human testes. (RNA was isolated from 5-40 mg testis biopsies. Patients gave informed consent for their collection and analysis.) We then searched those transcriptomes for the presence of 5 transcripts considered to be selectively expressed by SSCs (42). All SCO testes contained these transcripts. Shiraishi et al. (43) expanded our observation when they reported that immunocytochemical analysis of some SCO testes identified cells that express the germ cell-specific marker, DDX4.

**Figure 5:**
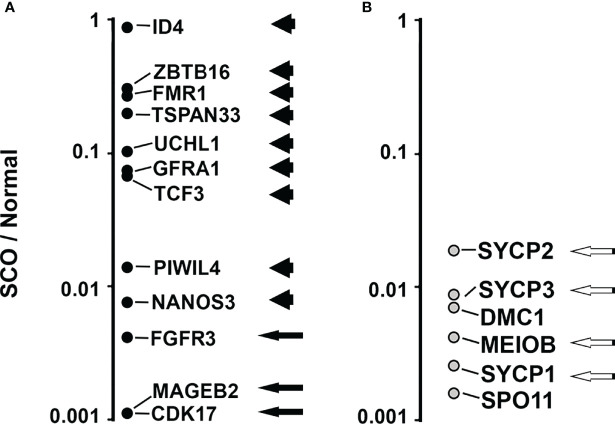
Comparing the abundance of 13 transcripts that are consensus markers of human SSCs **(A)** and 6 transcripts that are consensus markers of human pachytene spermatocytes **(B)** in the transcriptomes of 7 human SCO testes and 4 normal testes. All 19 consensus markers were identified in the transcriptomes of the 4 normal testes. Nine of the 13 SSC markers were identified in the transcriptomes of all 7 SCO testes (Panel **A**, arrowheads). Three SSC markers were present in the transcriptomes of 5 SCO testes (Panel **A**, black arrows). An additional SSC marker, NANOS2, was not present in the transcriptome of any SCO testes. (NANOS2 data are not shown in panel **(A)**. Four markers of pachytene spermatocytes, were present in all 7 SCO transcriptomes (Panel **B**, white on black arrows). DMC1 and SPO11 were present in 5 and 1 of the transcriptomes of SCO testes, respectively. To compare differences between SCO and normal testes in the abundance of each marker, we first normalized data by dividing the abundance of each marker in each transcriptome by the abundance of β actin mRNA in the same transcriptome. We then calculated the average normalized abundance of each marker in the transcriptomes of SCO testes and in the transcriptomes of normal testes. To illustrate the normalized abundance of 18 of the 19 markers in SCO testes (NANOS2 not shown), data for each SSC consensus marker **(A)** and each pachytene spermatocyte consensus marker **(B)** are presented as a ratio (SCO/Normal). Except for ID4, the abundance of each SSC marker is substantially lower in SCO testes than in normal testes. The median ratio (SCO/Normal) for the abundance of the 13 SSC markers was 0.07. The median ratio for abundance of the 6 pachytene spermatocyte markers was 0.004. Thus, some SCO testes contain SSCs, but few support the production of pachytene spermatocytes. The transcriptomes of normal and SCO human testes are described in Paduch et al. (42), and RNAseq data deposited in the NCBI dbGAP database, accession number: phs001777.v1.p1.

Since our analysis of the transcriptomes of SCO testes, several laboratories published the transcriptomes of every cell type in the human testes that were obtained by single cell RNA sequencing. We identified 13 transcripts that 3 different reports identify as selectively expressed by human SSCs (23, 44, 45). We reasoned that identification of these 13 SSC markers in SCO testes would further support the hypothesis that some SCO testes contain SSCs. All 13 markers were detected in the transcriptomes of the 4 normal testes. Nine were present in the transcriptomes of all 7 SCO testes (**Figure 5A**, arrowheads). Three were present 5 of these transcriptomes (**Figure 5A**, arrows). One consensus SSC marker, NANOS 2, was present in the transcriptomes of all 4 normal testes but in none of the SCO transcriptomes. This absence of NANOS2 mRNA in SCO testes might be explained by the facts that GDNF stimulates NANOS2 expression by mouse SSCs (46), and that the GDNF concentration in SCO testes is substantially lower than in normal human testes (5).

To illustrate the differences between SCO and normal testis in the average abundance of each SSC marker, we first normalized data by dividing the abundance of each SSC marker in each transcriptome by abundance of β-actin mRNA. We then calculated the average normalized abundance of each SSC marker in the transcriptomes of normal testes and in SCO testes. Finally, we divided the average normalized abundance in each marker in SCO testes by the average normalized abundance in normal testes (**Figure 5A**). Except for ID4, each SSC marker was substantially less abundant in SCO testes than in normal testes. The median abundance of the 13 SSC markers in SCO testes was 7% of normal.

We previously reported that in SCO testes the expression of 2 putative markers of pachytene spermatocytes were reduced to much greater extent than expression of putative SSC markers. To further support for this observation, we used the same strategy we used to compare abundance of each consensus SSC marker in SCO and normal testes. We first identified 6 consensus pachytene spermatocyte markers. All 6 were identified in two recent reports as selectively expressed by human pachytene spermatocytes, and proven to be essential for the fertility of male mice (23, 45, 47–52). These 6 markers are identified in **Figure 5B**. Four consensus markers were present in all 7 SCO testes (Black arrows, **Figure 5B**). DMC1 and SPO11 were present in the transcriptomes of 5 and 1 of the SCO testes, respectively (**Figure 5B**). To compare the abundance of each of the 6 consensus markers in SCO testes with their abundance in normal testes, we divided the average normalized abundance of each transcript in SCO testes by their average normalized abundance in normal testes (**Figure 5B**). The median abundance of these 6 markers in SCO testes was 0.4% of normal, which is much lower than the median abundance (7%) of SSC markers in the same transcriptomes. Thus, while some SCO testes contain SSCs, most do not give rise to pachytene spermatocytes.

### Reduced Expression of FGF2 and FGF8 in SCO Testes

**Figure 6:**
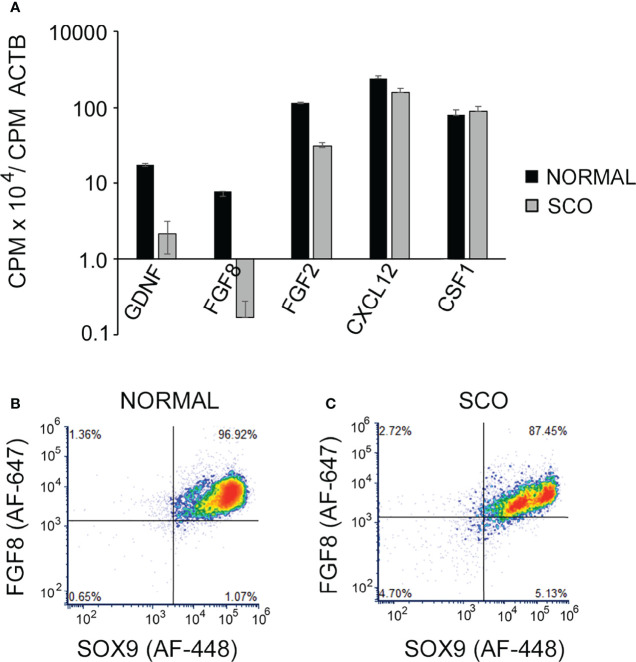
The abundance of GDNF, FGF8, GF2, CXCL12 and CSF1 mRNAs in the transcriptomes of human normal and SCO testes. **(A)** CPMs of each transcript in each of the transcriptomes of 4 normal and 7 SCO testes were normalized for CPM of β actin. Normalized data (mean + SEM) confirm that GDNF mRNA levels are markedly lower in SCO testes and reveal that expression of both FGF8 and FGF2 mRNA are also significantly reduced in SCO testes. **(B, C)** Fluorescence activated cell sorting was used to determine if human Sertoli cells express FGF8 and if this expression is significantly reduced in SCO testes. Single cell suspensions were prepared from normal and SCO testes, cells were immunolabeled for FGF8 and the human Sertoli cell-specific marker SOX9 (23) and FGF8 and SOX9 expression analyzed. Results, which are representative of 3 independent experiments, present FGF8 and Sox 9 expression by individual cells. This analysis demonstrates that normal human testes contain a single population of FGF8-expressing Sertoli cells. SCO testes contain 2 populations, and cells in the larger population contain less FGF8 than Sertoli cells in normal testes. Results are from: (42).

Studies of mice have identified numerous growth factors and chemokines that in addition to GDNF regulate SSC replication, differentiation and/or function. These include FGF8, FGF2, CXCL12 and CSF1. Mouse SSCs express FGFR1, a receptor for both FGF8 and FGF2 and Cre-mediated excision of this receptor from spermatogenic cells, results 24 months later in a significant decrease in numbers of GFRA1^+^ spermatogonia (53). *In vivo*, testicular overexpression of FGF8 causes numbers of GFRA1^+^spermatogonia to double within 15 days of virus injection, while injection of FGF2-containing microspheres stimulates formation of large clusters of these cells (53, 54). *In vitro*, CXCL12 stimulates proliferation and suppresses differentiation of SSCs (55). *Furthermore, in vivo*, this chemokine acts as a homing signal for SSCs, and, thus, may play an important role in the migration of SSCs into empty niches (56). *In vitro*, CSF1 stimulates self-renewing SSC replication, and transient depletion of testicular macrophages, a major CSF1, diminishes numbers of ZBTB16^+^ spermatogonia (55, 57). As human SSCs and/or progenitor spermatogonia express receptors for these growth factors, we examined the transcriptomes of normal and SCO testes to determine whether expression of any of these factors was markedly lower in human SCO testes (**Figure 6A**) (58, 59). As a control, we also examined the abundance of GDNF, to ensure that the RNAseq data replicated our previous results (See: **Figure 4**). It did. Furthermore, the transcriptomes of SCO testes revealed significantly reduced abundance of FGF2 and FGF8 mRNAs, but not of CXCL12 and CSF1 mRNAs. As the abundance of FGF8 mRNA in SCO testes was only 2% of control, we used FACs analysis to evaluate if this reduced expression reflected a specific deficit in Sertoli cell function. Result (**Figure 6B**) show in normal testes, FGF8 is expressed by a single population of Sertoli cells. In contrast, SCO testes contained two populations. One population contained markedly lower amounts of FGF8 than Sertoli cells in a normal testis (**Figure 6C**).

In summary most Sertoli cells in human SCO testes express abnormally low levels of GDNF as well as reduced levels of FGF2 and FGF8. Furthermore, some human SCO testes contain SSCs, though most do sustain production of pachytene spermatocytes. However, since a subset of the Sertoli cells contain normal amounts of GDNF, some of these cells may support the foci of active spermatogenesis present in some SCO testes (26).

### Why Do the SSCs in Human SCO Testes Give Rise to So Few Pachytene Spermatocytes?

As already discussed, a comparison of the transcriptomes of normal and SCO human testes suggests that in SCO testes few SSCs give rise to pachytene spermatocytes. The cause of this apparent maturation arrest might be intrinsic to the cells, themselves. Alternatively, it might result from inadequate testicular levels of extrinsic stimulators of progenitor spermatogonia differentiation. Kit ligand is one such stimulator (1), but as already discussed, KITL mRNA levels are elevated in SCO testes. However, there may be deficiencies in testis levels of a second stimulator, retinoic acid, for a preliminary study reported that testicular levels of 13-cis retinoic acid are lower in men with an abnormal semen analysis than in men with a normal one (60, 61). While we did not quantify testicular retinoic acid concentrations, we reasoned that if RA levels were normal in SCO testes, their transcriptomes should reveal normal expression of the enzymes that catalyze the 2-step conversion of retinol to retinoic acid (62). In the testis, the first and rate-limiting step, the conversion of retinol to retinal, is catalyzed by retinal dehydrogenase 10 (RDH10), and Cre-mediated excision of this gene in both Sertoli cells and germ cells of prepubertal mice results in maturation arrest of progenitor spermatogonia (63). The second step, the conversion of retinal to retinoic acid can be catalyzed by one of three different retinaldehyde dehydrogenases expressed in testes, ALDH1A, ALDH1A2 and ALDH1A3 (62). Human Sertoli and peritubular myoid cells express ALDH1A1, pachytene spermatocytes and round spermatids express ALDH1A2, and Sertoli cells and pachytene spermatocytes express ALDH1A3 (64).

**Figure 7:**
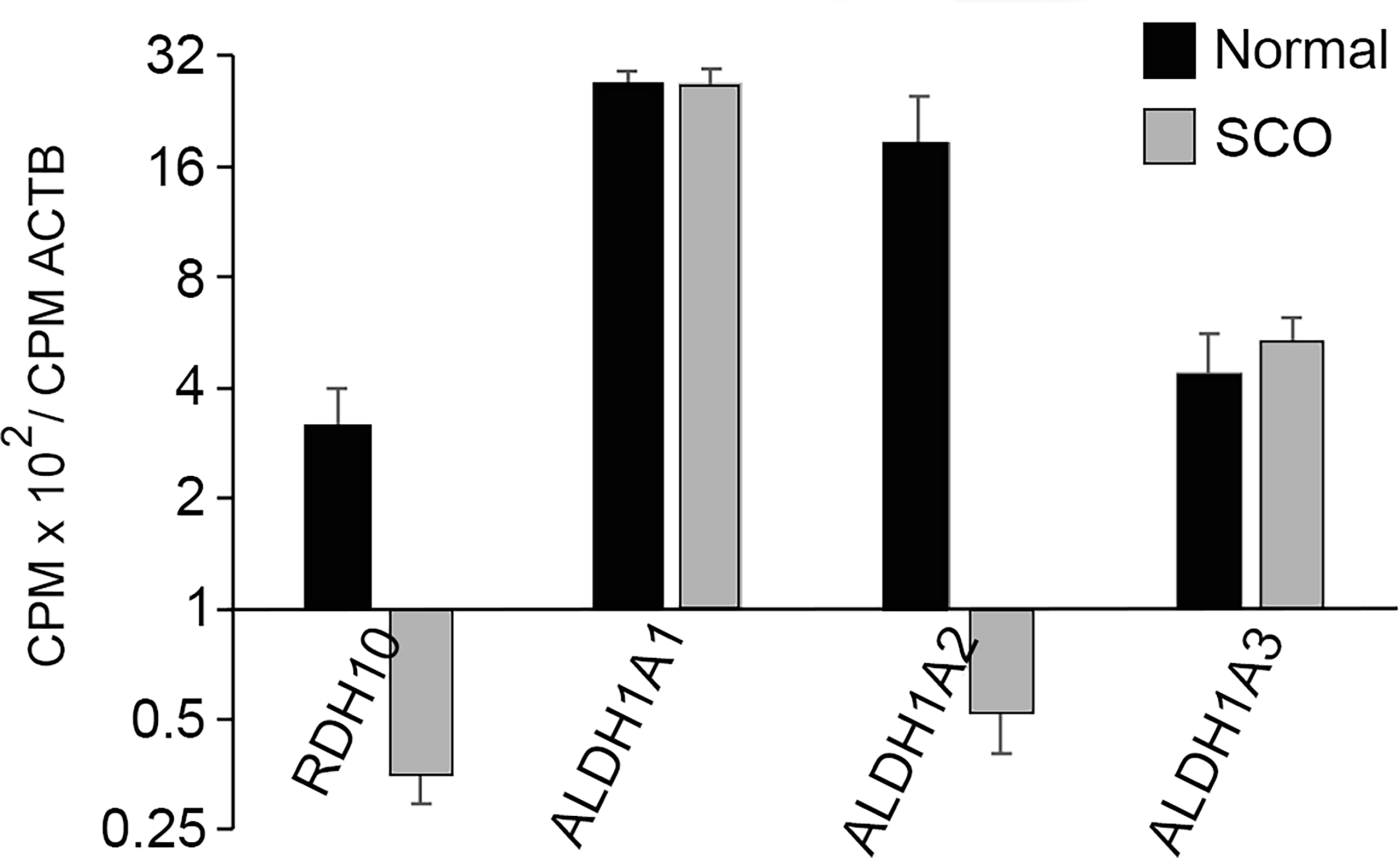
The abundance in the transcriptomes of normal and SCO human testes of transcripts that encode the enzymes catalyzing the two-step conversion of retinol to retinoic acid. CPMs of each transcript in each of the transcriptomes of 4 normal and 7 SCO testes were normalized for CPM of β actin. Normalized data (mean + SEM) demonstrate that expressions of RDH10 and ALDH1A2 are substantially reduced in human SCO testes.

A comparison of the transcriptomes of normal and SCO testes reveals that expression of RDH10 in SCO testes is only 10% of normal (**Figure 7**). However, expressions of ALDH1A1 and ALDH1A3 are normal, while expression of ALDH1A2 is reduced, as would be expected for a gene expressed by germ cells (**Figure 7**). As retinoic acid stimulates differentiation of progenitor spermatogonia, as RDH10 is the rate limiting step in the conversion of retinol to retinoic acid, and as expression of this enzyme is markedly reduced in SCO testes, a comparison of retinoic acid concentrations in normal and SCO human testis is warranted.

## Hypothesis 3: A Mouse SSCs Pool That Has Been Partially Depleted Due to Inhibition of GDNF Signaling Will Rebuild if GDNF Signaling Is Restored

The fact that most Sertoli cells in human SCO testes express low amounts of GDNF raised the question of whether a pool of SSCs that has been depleted due to inadequate GDNF stimulation would rebuild if adequate stimulation to the remaining stem cells was restored. We took the first step to answering this question by use of our mouse model. We injected mice for 9 days with 1NAPP1 and sacrificed mice 2-4 days or 2 months later after injections ceased. Loss and restoration of SSCs were evaluated using two different morphological approaches. The first enumerated cells that co-expressed two different SSC markers, ID4-GFP and GFRA1. The second counted seminiferous tubules that 2 months after treatment were characterized as exhibiting normal spermatogenesis, incomplete spermatogenesis, or SCO syndrome (absence of all germ cells). Tubules exhibiting incomplete spermatogenesis contained 2-4 generations of germ cells, rather the normal 4 to 5 generations (33).

**Figure 8:**
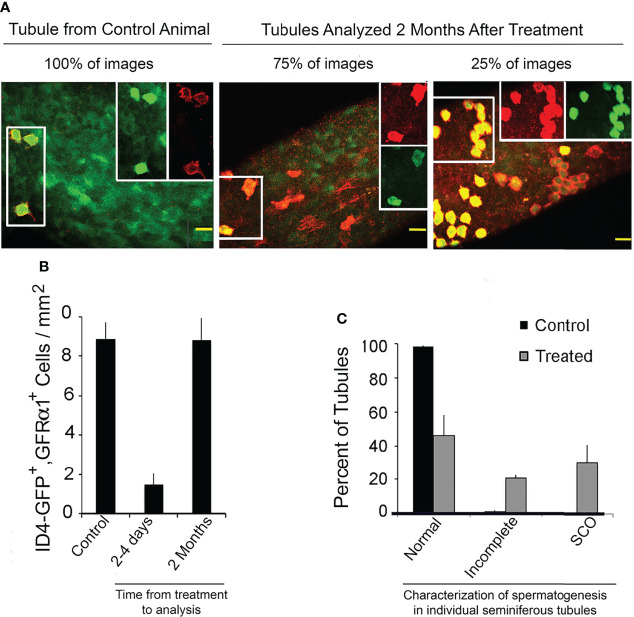
A mouse SSCs pool that has been partially depleted due to inhibition of GDNF signaling will rebuild if GDNF signaling is restored. Mice used for this experiment were from the same liters that provided mice for enumeration of transplantable SSCs (See: **Figure 2A**). Ret(V805), Rosa 26 ^+/-^, ID4-GFP ^+/-^ mice were injected for 9 days with vehicle (control) or with 1NAPP1, tubules were collected 2 to 4 days or 2 months thereafter and GFRα1-expressing cells detected by immunocytochemistry. Tubules were imaged by confocal microscopy and SSCs were defined as co-expressing GFRα1 (red fluorescence) and ID4-GFP (green fluorescence). Intact testes from additional control and treated mice (n = 3/group) were collect 2 months after the last injections and process for light microscopy as described for **Figure 3**. Spermatogenesis in each tubule cross section was examined. **(A)** Confocal micrographs of tubules of control mice, and of tubules from mice sacrificed 2 months after treatment with 1NAPP1. Cells that expressed both GFRα1 and GFP are outlined by a box on the left had side of each image. Separate red and green channels for the same cells are shown in the boxes on the right side. GFRα1^+^, ID4-GFP^+^ cells were identified as A_s_ spermatogonia in all images of tubules from control mice, in all images of tubules from mice sacrificed 2-4 days after treatment and in 75% of the images of tubules collected 2 months after treatment. In the other 25% of those images, GFRα1^+^, ID4-GFP^+^ cells were present as chains or clusters of cells. **(B)** Numbers of GFRα1^+^, ID4-GFP^+^ spermatogonia per mm^2^ of tubule in control mice, in mice sacrificed 2-4 days after treatment and in mice sacrificed 2 months after treatment. Data are presented as mean + SEM for 5 control animals and for 3 mice at each of the two time points. **(C)** Characterization of spermatogenesis in seminiferous tubules of control mice and of mice sacrificed 2 months after treatment (n = 3/group). Tubules were characterized as having normal spermatogenesis, incomplete spermatogenesis (containing 2-4 generations of germ cells) or lacking all germ cells and thus exhibiting SCO syndrome. Normal or incomplete spermatogenesis was observed in 71% of tubules of treated mice. Taken together, these results demonstrate that when GDNF signaling is inhibited for 9 days and then restored, the depleted SSC pool is partially rebuilt. Results are from: (16).

All images of control tubules, all images of tubules collected 2-4 days after treatment and 75% of the images from tubules collected 2 months after treatment, showed ID4-GFP^+^, GFRA1^+^ cells to be A_s_ spermatogonia (**Figure 8A**), another morphological characteristic of SSCs (1). However, 2 months after treatment, 25% of the images revealed clusters or chains of ID4-GFP^+^, GFRA1^+^ spermatogonia. We suggest that these clusters or chains exist at the interface between areas of tubules with refilled stem cell niches and areas with empty niches. Morphometric analysis demonstrated that 9 days of treatment reduced numbers of ID4-GFP^+^, GFRA1^+^ cells by 84%. Two months later, their numbers were normal (**Figure 8B**).

Histological analysis demonstrated that 2 months after treatment, 71% of tubule cross sections exhibited either normal or incomplete spermatogenesis (**Figure 8C**). The remaining 29% of the tubules were characterized as SCO.

Taken together, those data demonstrate that an SSCs pool that has been substantially depleted due to inadequate GDNF stimulation will substantially rebuild if the remaining SSCs are provided adequate GDNF stimulation.

## Potential Therapy for Some Infertile Men With SCO Syndrome

The data presented in this review prove that GDNF is essential for maintaining a normal pool of SSCs in an adult mouse testis. We and others have demonstrated that most Sertoli cells in human SCO testes express abnormally low levels of GDNF, and analysis of GDNF^+/-^ mice indicate that such levels are insufficient to sustain a normal stem cell pool (4, 43). Our data also demonstrated that the few SSCs remaining after 9 days of inhibited GDNF signaling will partially rebuild the stem cell pool within 2 months if adequate GDNF stimulation resumes. Taken together, these observations suggest that increasing the concentration of GDNF in a human SCO testis might stimulate the few SSCs they contain to increase in numbers, whether those stem cells are in areas of a tubule without other spermatogenic cells or whether they are present in foci of active spermatogenesis. As GDNF stimulates the migration of SSCs (65), an increase in GDNF testicular concentration, might also stimulate SSCs to migrate to empty niches and potentially seed active spermatogenesis in a previous barren area of tubule. Such an increase in the size or number of spermatogenic foci would increase the probability of successful sperm retrieval by micro-TESE.

Given the above considerations, how could GDNF be developed as therapy for SCO syndrome? A potential approach is suggested by methods that are being developed to stimulate repair injured neurons by local administration of GDNF. Three different methods have been described for this local administration: Driving *de novo* expression of GDNF at the site of injury by injection of non-replicating virus that encode GDNF, implantation of GDNF-containing microspheres, and implantation of cells that express recombinant GDNF (66–70). In the last approach, GDNF-secreting cells are encapsulated in matrices that protected those cells from immune attack, while allowing free diffusion of proteins to and from the cells.

It is well established that chronically increasing the concentration of testicular GDNF in rodent testes substantially suppresses SSC differentiation, causing their substantial overaccumulation (4, 69, 71). Eventually the structure of the seminiferous epithelium is disrupted, and spermatogenesis fails. As noted by Sharma et al. (71) the likely reason for this overaccumulation is that normally, the expression of GDNF by rodent Sertoli cells changes more than 10-fold as the adjacent germ cells progress through the stages of the cycle of the seminiferous epithelium (28, 71). We have proposed that this cycle of GDNF expression results in SSC replication at some stages of the cycle and SSC differentiation at others (28). We anticipate that successful use of GDNF as therapy for SCO syndrome will require that the therapy cycle the testes between periods of elevated GDNF concentration and periods of lower concentration. This goal might he achieved by implanting SCO testes with encapsulated cells that drive GDNF expression *via* a bacterial Tet operon (72). By interspersing days of oral tetracycline administration with days of placebo administration, a cycle of testis GDNF levels might be achieved.

We recognize that development of this proposed therapy will take much effort, time, and resources. Defining the proper number of implanted cells will be essential, as well as their placement within a testis. Developing an efficacious tetracycline dosing schedule will also be critical. Moreover, as FGF2 and FGF8 expression is also abnormally low in SCO testes, implants of cells expressing one or both of growth factors also may be required. However, the successful development of this new therapy may allow some infertile men with SCO syndrome to father their own children.

## Author Contributions

The author confirms being the sole contributor of this work and has approved it for publication.

## Funding

Supported by HD 1RO1HD074542.

## Conflict of Interest

The author declares that the research was conducted in the absence of any commercial or financial relationships that could be construed as a potential conflict of interest.

## Publisher’s Note

All claims expressed in this article are solely those of the authors and do not necessarily represent those of their affiliated organizations, or those of the publisher, the editors and the reviewers. Any product that may be evaluated in this article, or claim that may be made by its manufacturer, is not guaranteed or endorsed by the publisher.
